# Risk score constructed with neutrophil extracellular traps-related genes predicts prognosis and immune microenvironment in multiple myeloma

**DOI:** 10.3389/fonc.2024.1365460

**Published:** 2024-06-11

**Authors:** Gongzhizi Gao, Rui Liu, Dong Wu, Dandan Gao, Yang Lv, Xuezhu Xu, Bingjie Fu, Zujie Lin, Ting Wang, Aili He, Ju Bai

**Affiliations:** ^1^ Department of Hematology, The Second Affiliated Hospital of Xi’an Jiaotong University, Xi’an, China; ^2^ National-Local Joint Engineering Research Center of Biodiagnostics & Biotherapy, The Second Affiliated Hospital of Xi’an Jiaotong University, Xi’an, China; ^3^ Xi’an Key Laboratory of hematological diseases, Xi’an, China

**Keywords:** multiple myeloma, neutrophil extracellular traps, risk score, nomogram, immune microenvironment, drug sensitivity

## Abstract

**Background:**

Multiple myeloma (MM) exhibits considerable heterogeneity in treatment responses and survival rates, even when standardized care is administered. Ongoing efforts are focused on developing prognostic models to predict these outcomes more accurately. Recently, neutrophil extracellular traps (NETs) have emerged as a potential factor in MM progression, sparking investigation into their role in prognostication.

**Methods:**

In this study, a multi-gene risk scoring model was constructed using the intersection of NTEs and differentially expressed genes (DEGs), applying the least absolute shrinkage and selection operator (LASSO) Cox regression model. A nomogram was established, and the prognostic model’s effectiveness was determined via Kaplan-Meier survival analysis, receiver operating characteristic (ROC) curve, and decision curve analysis (DCA). The ESTIMATE algorithm and immune-related single-sample gene set enrichment analysis (ssGSEA) were employed to evaluate the level of immune infiltration. The sensitivity of chemotherapy drugs was assessed using the Genomics of Drug Sensitivity in Cancer (GDSC) database. Ultimately, the presence of the detected genes was confirmed through quantitative real-time polymerase chain reaction (qRT-PCR) analysis in MM cell specimens.

**Results:**

64 NETs-DEGs were yielded, and through univariate Cox regression and LASSO regression analysis, we constructed a risk score composed of six genes: CTSG, HSPE1, LDHA, MPO, PINK1, and VCAM1. MM patients in three independent datasets were classified into high- and low-risk groups according to the risk score. The overall survival (OS) of patients in the high-risk group was significantly reduced compared to the low-risk group. Furthermore, the risk score was an independent predictive factor for OS. In addition, interactions between the risk score, immune score, and immune cell infiltration were investigated. Further analysis indicated that patients in the high-risk group were more sensitive to a variety of chemotherapy and targeted drugs, including bortezomib. Moreover, the six genes provided insights into the progression of plasma cell disorders.

**Conclusion:**

This study offers novel insights into the roles of NETs in prognostic prediction, immune status, and drug sensitivity in MM, serving as a valuable supplement and enhancement to existing grading systems.

## 1 Introduction

Multiple myeloma is a plasma cell malignancy characterized by abnormal proliferation of monoclonal plasma cells in the bone marrow, which secrete large amounts of monoclonal immunoglobulin. It is the second most common hematologic malignancy (1). Despite of advancement in treatment options in recent years, MM remains biologically complex with heterogeneous prognosis. Therefore, it is crucial to identify reliable prognostic indicators and establish corresponding predictive models for patients with MM.

Tumor microenvironment (TME) is a complex ecosystem composed of various cells and molecules that play a crucial role in the initiation, progression, and metastasis of tumors. This microenvironment not only includes tumor cells, but also immune cells, stromal cells, as well as an interactive network constructed by extracellular matrix and peripheral blood vessel (2). These molecules and cells interact with each other to shape a multifaceted biological scene, providing support for tumor growth and metabolism, as well as creating conditions for immune evasion and facilitating distant metastasis (3, 4). Among TME, immune cells play a particularly critical role, influencing tumor growth and metabolism, and participating in the formation of tumor immune escape mechanisms. The bone marrow, as the main site of onset for MM, provides a favorable environment for the growth and spread of MM cells (5). As the most abundant immune cells in the tumor microenvironment, neutrophils participate in the process of tumors by releasing NETs (6). NETs are web-like structures composed of DNA, histones, and granule enzymes, responsible for trapping and killing extracellular pathogens (7). The process of NETs being released by neutrophils is called NETosis, a cell death pathway reported to be distinct from apoptosis, phagocytosis-induced cell death, and necrosis (8). Based on the fate of neutrophils, NETosis can be divided into two types: suicidal NETosis and vital NETosis (9). Increasing research has indicated that NETs play a significant role in many diseases (10). Interestingly, NETs are involved in the initiation and progression of tumors, with dual roles (pro-tumor and anti-tumor) varying according to the type of cancer, different stages of cancer development, statuses of the immune system, and tumor microenvironment conditions (11, 12). In the realm of hematologic disorders, studies have indicated that neutrophils from chronic lymphocytic leukemia patients exhibit an increased capacity to release NETs (13, 14). NETs could increase the burden of bleeding by damaging endothelial cells in acute promyelocytic leukemia (15). In addition, higher levels of NETs in plasma and tumor tissues were associated with a dismal outcome in patients with diffuse large B-cell lymphoma (16). However, the role of NETs in MM remains unclear.

To further understand the relationship between MM and NETs, we retrieved DEGs between MM patients and normal individuals from the Gene Expression Omnibus Database (GEO). Gene Ontology (GO) enrichment analysis indicated these DEGs were involved in neutrophil and leukocyte-associated pathways. Based on this, we constructed a DEGs-NETs network and selected six core genes to compose a risk score. Then, we examined the association between the risk score and clinical indicators and developed a nomogram using multivariate Cox regression analysis. Finally, we verified roles of the risk score in immune response and drug resistance. In summary, our study established a six-gene risk score and a prognostic model, providing novel insight into the survival assessment, immune microenvironment and drug resistance in MM.

## 2 Materials and methods

### 2.1 Databases and data preprocessing

We downloaded multiple GEO microarray datasets, including the microarray data and clinical information of samples from GSE6477, GSE136337, GSE57317 and GSE2658 from the GEO database (https://www.ncbi.nlm.nih.gov/geo/). Among them, the GSE6477 dataset (GPL96) which sample type is bone marrow, involves 22 cases of Monoclonal Gammopathy of Undetermined Significance (MGUS), 24 cases of Smoldering Multiple Myeloma (SMM), 73 cases of Newly Diagnosed Multiple Myeloma (NDMM), 28 cases of Relapsed and/or Refractory Multiple Myeloma (RRMM) samples and 15 cases of control group samples. NDMM and the control group were used for differential expression analysis. The GSE136337 dataset (GPL27143) contains 426 bone marrow samples of NDMM, which were used to construct risk score and nomogram. The GSE57317 dataset (GPL570) and the GSE2658 dataset (GPL570) contain 55- and 559-MM bone marrow samples respectively to verify the risk score. The maximum expression level of gene symbols identified by multiple probes was used for calculation. **Supplementary Table 1** shows the baseline information of the training and validation cohorts.

### 2.2 Identification and enrichment analysis of candidate genes

DEGs between NDMM samples and normal samples were identified by using the “limma” R package (version 3.52.2) (17). The heatmap was performed by the “ComplexHeatmap” R package (version 2.13.1) (18). We set genes with padj < 0.05 and |logFC| > 0.4 as DEGs to control the number of DEGs and reduce the possibility of false-positive results. The NETs-related genes originate from previously published literature (19–22) (**Supplementary Table 2**). Candidate genes were obtained by taking the intersection of DEG and NETs genes. The results were then visualized using the ggvenn R package (version 0.1.9). We conducted GO and KEGG enrichment analyses to evaluate the potential functions of DEGs-NETs. After ID conversion of the input molecular list, the “clusterProfiler” R package (version 4.4.1) was used for enrichment analysis to obtain the gene set enrichment results. p-value < 0.05 was considered significantly enriched. GO and KEGG pathway enrichment results were visualized using the enrichplot R package (version 1.8.1).

### 2.3 Screening of prognostic genes, construction and validation of risk score

Using the R “survival” package (version 3.4.0), we performed univariate Cox regression on the GSE136337 dataset to evaluate the OS-related gene (HR ≠ 1, p < 0.05). Subsequently, we continued to use the “glmnet” R package (version 4.1.1) in the GEO136337 dataset to generate prognostic features through the LASSO Cox regression method. The risk score was calculated as follows: Risk Score = (-0.175709931 × expr(CTSG)) + (0.045561562 × expr(HSPE1)) + (0.346091322 × expr(LDHA)) + (-0.008019573 × expr(MPO)) + (-0.257014272 × expr(PINK1)) + (-0.062861009 × expr(VCAM1)). After obtaining the prognostic genes and risk scores, the expression of MM samples was calculated. Using the “Survminer” R package (version 0.4.9), patients with MM were classified into low-risk and high-risk groups based on risk score, and survival differences between the two groups were compared. Additionally, the sensitivity and specificity of the risk score were evaluated using the “timeROC” R package. In addition, the performance of the risk model was also validated in the GSE557317 and GSE2658 datasets. To explore the relationship between risk scores and clinical features, six clinical features (age, gender, β2-microglobulin (β2M) level, lactate dehydrogenase (LDH) level, presence of high-risk cytogenetics, and Revised International Staging System (R-ISS) stage) were used to conduct a group comparison between high and low-risk groups on the GEO136337 dataset.

### 2.4 Development and validation of nomogram

We developed a predictive nomogram that combines the risk score from the prediction model with clinical features to identify independent prognostic factors among risk score, age, gender, β2M, LDH, high-risk cytogenetics, and R-ISS stage. A p-value < 0.05 was used to select survival-related clinical variables through univariate Cox analysis. Subsequently, a multivariate survival analysis was performed to create the nomogram. The nomogram predictions were plotted using calibration curves based on the measured rates. We used R packages “rms” (version 6.3–0), “stdca,” and “timeROC” to plot the nomogram, calibration curve, DCA plot, and ROC curve.

### 2.5 Gene set enrichment analysis

To explore the functions and pathways associated with the risk groups, a differential expression analysis was performed on the genes expressed in the high and low-risk groups on the dataset GSE136337.Gene Set Enrichment Analysis (GSEA) is a computational strategy used to analyze whether a prior-defined set of genes is enriched in a typical signaling pathway (https://www.gsea-msigdb.org/gsea/index.jsp). padj < 0.05 and false discovery rate (FDR) q-value < 0.05 were considered significantly enriched.

### 2.6 Assessment of immune microenvironment

Based on the transformed gene expression data, the “estimate” R package (23) was used to calculate the ESTIMATE score, immune score, and stromal score. Additionally, the “GSVA” R package was used to estimate the infiltration abundance of various immune cells. The effect of immune checkpoints was explored by analyzing the differential expression of immune checkpoints in two risk groups of MM samples. The differences in immune checkpoints and immune cells between the high and low-risk groups were visualized through grouped comparison charts.

### 2.7 Drug sensitivity prediction

To achieve precision therapy based on NETs-related characteristics and to identify potential drugs for MM, we utilized the genomic data from the GDSC (https://www.cancerrxgene.org/) to predict chemotherapeutic responses. The R package “pRRophetic” calculates (24) half-maximal inhibitory concentration (IC50) values to reflect drug response of 138 antitumor agents.

### 2.8 TISCH analysis

We utilized the TISCH resource, a single-cell RNA sequencing online repository (Tumor Immune Single-cell Hub, accessible at http://tisch.comp-genomics.org/), to assess the presence of potential tumor antigens within immune cells that have penetrated the bone marrow. Data set GSE16180 within the TISCH platform was categorized into 9 principal cell categories, which enabled the visualization of specific gene expression patterns across different types of immune cells.

### 2.9 Cell culture

Human multiple myeloma cell lines MM.1S, PMI-8226, NCI-H929, OPM-2, U266, ARD, and KMS-11 were purchased from the ATCC U.S. Typical Biological Resources Repository (ATCC Cell Bank). The above cells were cultured by RPMI1640 mixed medium containing 10% fetal bovine serum (FBS), penicillin 100U/ml and streptomycin 100μg/m1 in a special cell incubator at 37°c and CO2 concentration of 5%.

### 2.10 RNA extraction and quantitative real-time PCR

To quantify gene expression profiles, we performed qRT-PCR on MM cell lines. Total RNA was extracted from the samples utilizing TRIzol reagent (Thermo Fisher Scientific, USA) following the manufacturer’s protocol. The purity and concentration of RNA were measured spectrophotometrically. Complementary DNA (cDNA) synthesis was accomplished using the PrimeScript™ RT Master Mix (Takara Bio, USA) according to the provided instructions. Subsequent amplification reactions were set up using TB Green® Premix Ex Taq (Takara Bio, USA) in a real-time PCR system. Each sample was assessed in triplicate to ensure accurate quantification. We selected GAPDH as a housekeeping gene for normalization. The Cycle Threshold (CT) values for the 6 genes and the reference were recorded for each sample. The relative expression levels of the target genes were calculated using the 2^^-ΔΔCt^ method. Seven MM cell lines and control groups were tested using holistic tests (one-way ANOVA) and multiple hypothesis tests (Tukey HSD postmortem tests). Details of the primer sequences employed for qRT-PCR were provided in **Supplementary Table 3**.

### 2.11 Statistical analysis

Statistical analysis was performed using R Studio (version 4.2.1). Fisher’s exact test and chi-squared test were used to investigate whether there are significant differences in clinical characteristics between the high-risk and low-risk groups. For comparing continuous variables with non-normal distributions, we employed the Wilcoxon rank-sum and Kruskal-Wallis tests. Data visualization was conducted using the R “ggplot2” package (version 3.3.6). All statistical tests were two-sided, and statistical significance was defined as p < 0.05.

## 3 Results

### 3.1 Identification of NETs-DEGs

**Figure 1:**
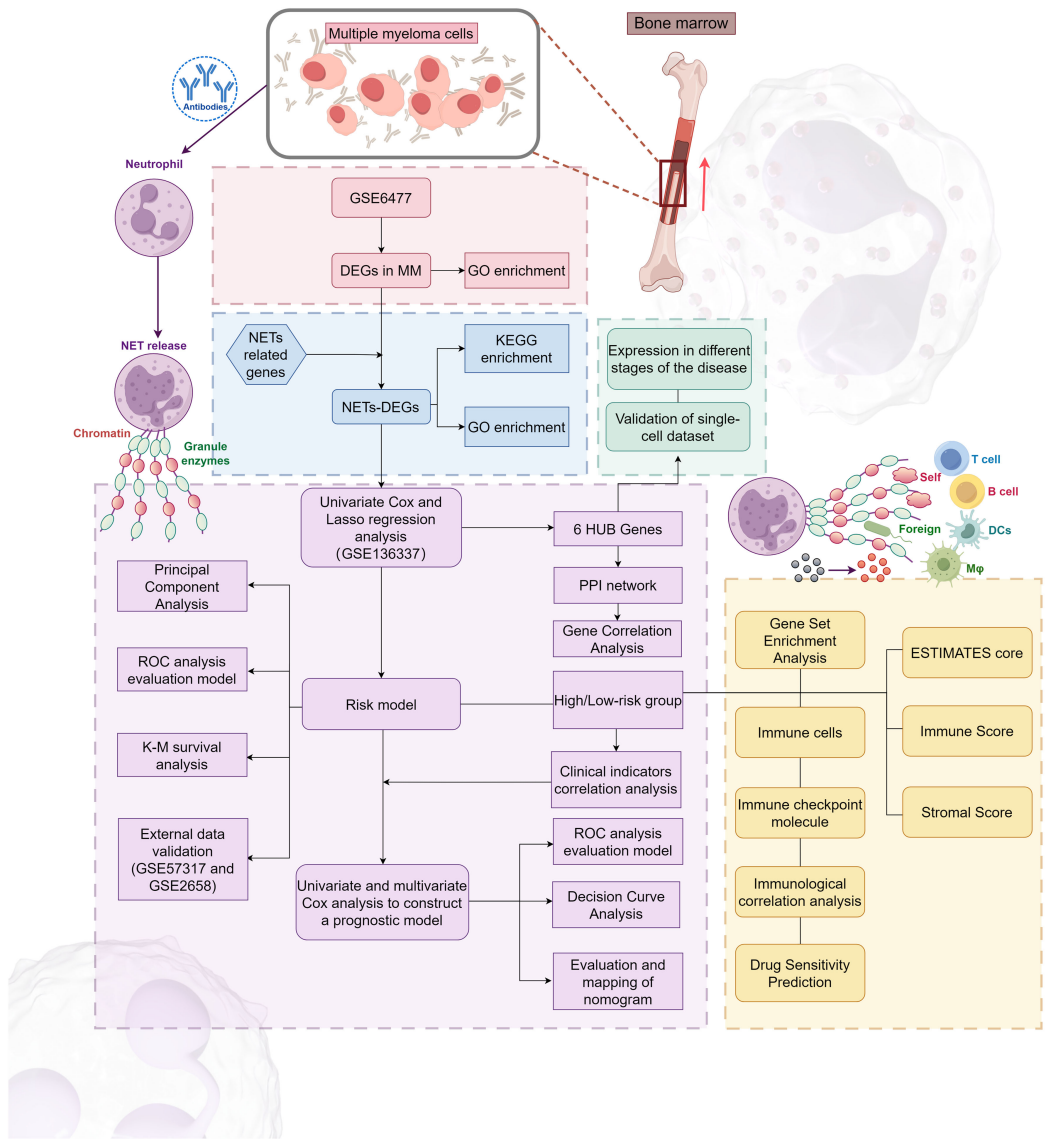
The flowchart of this study.

**Figure 2:**
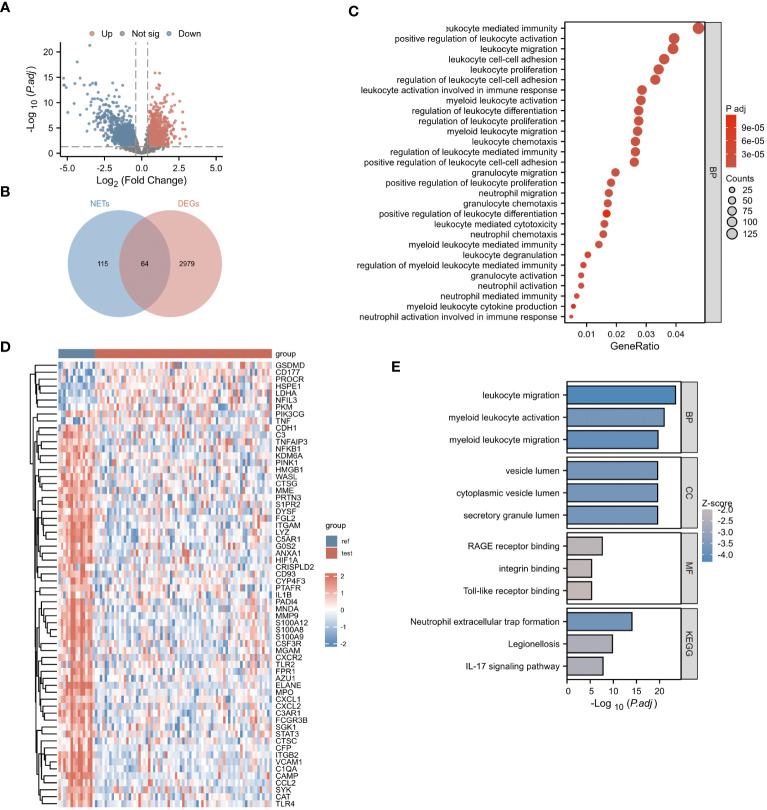
Identification of NETs-DEGs. **(A)** The volcano plot showed differentially expressed genes. Upregulated and downregulated genes were represented in red and blue, respectively. **(B)** The bubble plot illustrated the GO analysis of differentially expressed genes. **(C)** The Venn diagram displayed the overlap between differentially expressed genes and NETs-related genes. **(D)** The heatmap presented the DEGs-NETs genes. **(E)** The GO-KEGG analysis of DEGs-NETs genes (BP, biological process; CC, cellular component; MF, molecular function; KEGG, Kyoto Encyclopedia of Genes and Genomes), the color of the bar chart represents the significance level. The larger the absolute value of z-score and the deeper the color indicate the higher significance.

Our research process is outlined in **Figure 1** (By Figdraw.). Initially, we identified DEGs between patients with NDMM and healthy individuals from the GSE6477 dataset, as shown in **Figure 2A**. We found that among the DEGs, 1690 genes were up-regulated and 1353 genes were down-regulated. GO analysis of these DEGs revealed significant enrichment (p < 0.001) in pathways related to leukocyte-mediated immunity, myeloid leukocyte migration, neutrophil chemotaxis, neutrophil migration, and granulocyte chemotaxis (**Figure 2B**). Neutrophils, as the most abundant leukocytes in the human body, clear pathogens through phagocytosis and degranulation. Since these DEGs were associated with neutrophils, and the role of NETs has been found in various tumors in recent years (11), we further observed the relationship between DEGs and NETs. We performed an intersection analysis between the DEGs and genes known to be associated with NETs using a Venn diagram, which resulted in the identification of 64 DEGs-NETs, as shown in **Figure 2C**. **Figure 2D** presents a heatmap comparing the expression levels of these DEGs-NETs in healthy individuals and NDMM patients. Our analysis indicated lower expression levels of NETs-related genes in NDMM patients compared to healthy individuals. Subsequently, we conducted a combined GO-KEGG analysis of the DEGs-NETs, incorporating LogFC values (**Figures 2E**). This analysis unveiled significant enrichment in pathways related to leukocyte migration, myeloid leukocyte activation, myeloid leukocyte migration, vesicle lumen, cytoplasmic vesicle lumen, secretory granule lumen, RAGE receptor binding, Toll-like receptor binding, lipopolysaccharide binding, neutrophil extracellular trap formation, Legionnaires’ disease, and the IL-17 signaling pathway. These results showed that 64 DEGs-NETs had correlation with leukocyte migration and activation, as well as the formation of neutrophil extracellular traps.

### 3.2 Selection of hub genes and construction of the NETs-related risk score

**Figure 3:**
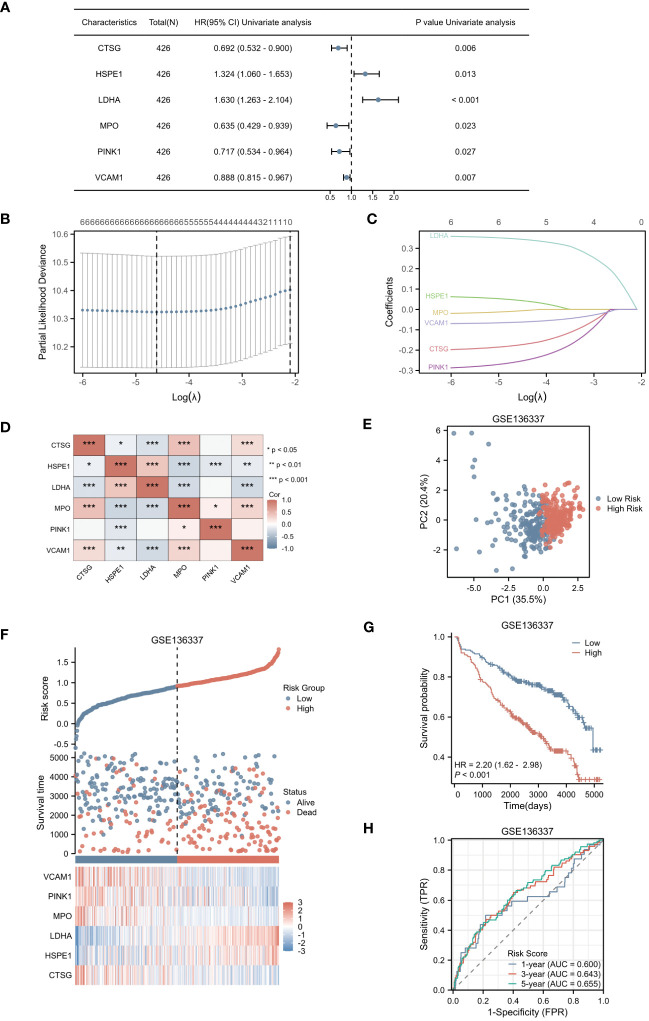
Selection of Hub Genes and Construction of the NETs-related Risk Score. **(A)** The forest plot displayed 6 genes with significant p-values (<0.05) after single-factor COX regression. **(B)** LASSO regression selected the best predictive variables through 10-fold cross-validation. **(C)** The LASSO coefficient trajectories of the 6 genes were plotted. **(D)** Pairwise correlation analysis was performed on the six genes. **(E)** The PCA plot of the high-risk and low-risk groups was created. **(F)** The risk factor plot between the high-risk group and the low-risk group was constructed. **(G)** KM curves for the high-risk and low-risk groups were generated. **(H)** The ROC curve of the overall survival time over time in the training set was drawn. *p<0.05; **p<0.01; ***p<0.001.

In our study, we initially identified 64 genes from the training set GSE136437. Subsequently, through univariate Cox regression analysis, we narrowed down this set to six prognostic genes: CTSG, HSPE1, LDHA, MPO, PINK1 and VCAM1 (as shown in **Figure 3A**). Among these genes, CTSG, MPO, PINK1 and VCAM1 exhibited protective effects (HR < 1), while HSPE1 and LDHA were classified as risk genes (HR > 1). To validate the expression of the six genes included in the risk score, we utilized the TISCH database to examine their expression in MM cells within the single-cell dataset GSE16180, as shown in **Supplementary Figure 1**. We discovered that HSPE1 and LDHA were expressed at higher levels in malignant cells, consistent with our preliminary findings that HSPE1 and LDHA are overexpressed in MM patients. As shown in **Supplementary Figure 2**, in the GSE6477 dataset representing different stages of MM development (MGUS, SMM, NDMM, RRMM), HSPE1 and LDHA exhibited gradually increasing expression, while CTSG, MPO, PINK1 and VCAM1 showed gradually decreasing expression. Therefore, these six genes not only predicts the prognosis of MM patients but may also provide insights into the diagnosis and disease progression stages in MM patients.

Utilizing LASSO regression, we developed a NETs-related risk score based on the expression levels of these six genes (**Figures 3B, C**). To further understand the interactions among these genes, we constructed a correlation heatmap for the six genes. Notably, strong positive correlations were observed between MPO and CTSG, as well as between HSPE1 and LDHA, while a strong negative correlation was observed between MPO and HSPE1 (**Figure 3D**). After excluding two patients due to missing survival data, the remaining patients were stratified into high-risk (N=212) and low-risk (N=212) groups based on their risk scores. Principal Component Analysis (PCA) effectively captured the variations between samples, as evidenced by the distances between the points in **Figure 3E**. The PCA clearly separated the high-risk and low-risk groups based on the expression profiles of the six prognostic genes. The distribution of risk scores in the training cohort indicated that patients with higher risk scores had poorer prognoses (**Figure 3F**). Kaplan-Meier survival analysis revealed a statistically significant difference in OS between the high-risk and low-risk groups, with the OS being significantly shorter in the high-risk group (p < 0.001) (**Figure 3G**). We evaluated the predictive performance of our model using time-dependent ROC curves. The Area Under the Curve (AUC) values for 1-year, 3-year, and 5-year OS were 0.600, 0.643, and 0.655, respectively, demonstrating the reliability of our model (**Figure 3H**).

### 3.3 Validation of risk score in two independent datasets

**Figure 4:**
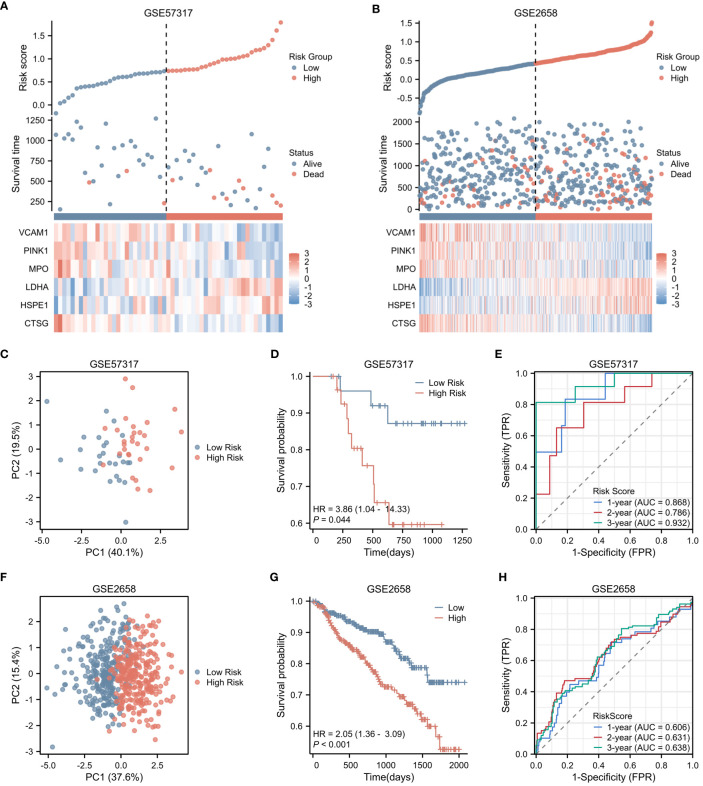
Validation of Risk Score in Two Independent Datasets. **(A)** Risk factor plot for the high-risk and low-risk groups in the validation cohort GSE57317. **(B)** Risk factor plot for the high-risk and low-risk groups in the validation cohort GSE2658. **(C)** PCA plot for the high-risk and low-risk groups in GSE57317. **(D)** Kaplan-Meier curves for the high-risk and low-risk groups in GSE57317. **(E)** Time-dependent ROC curves for overall survival in GSE57317. **(F)** PCA plot for the high-risk and low-risk groups in GSE2658. **(G)** Kaplan-Meier curves for the high-risk and low-risk groups in GSE2658. **(H)** Time-dependent ROC curves for overall survival in GSE2658.

In the validation cohorts derived from GSE57317 and GSE2658, patients were stratified into high-risk (N=28 and 280, respectively) and low-risk (N=27 and 279, respectively) groups, based on the median value of the calculated risk score. This stratification is clearly illustrated in **Figures 4A** and **B**. Within the validation cohort of GSE57317, PCA effectively discriminated between the high-risk and low-risk groups, with distinct clustering observed based on the expression profiles of the six prognostic genes (**Figure 4C**). Kaplan-Meier survival analysis revealed a statistically significant difference in OS between these two risk groups (p < 0.05) (**Figure 4D**). The predictive performance of our model was further evaluated using time-dependent ROC curves. The AUC values for 1-year, 2-year, and 3-year OS were 0.868, 0.786, and 0.932, respectively, demonstrating reasonable discriminatory ability (**Figure 4E**). Similarly, in the validation cohort of GSE2658, PCA analysis exhibited clear separation between the high-risk and low-risk groups based on the expression levels of the six genes (**Figure 4F**). Kaplan-Meier analysis showed a more pronounced difference in OS between the two groups, with a significantly shorter survival observed in the high-risk group (p < 0.001) (**Figure 4G**). The prognostic accuracy of our model was again confirmed using time-dependent ROC curves, yielding AUC values of 0.606, 0.631, and 0.638 for 1-year, 2-year, and 3-year OS, respectively (**Figure 4H**). These results collectively underscore the robustness and generalizability of the risk score across different patient cohorts.

### 3.4 Correlation between risk score and clinical characteristics

**Figure 5:**
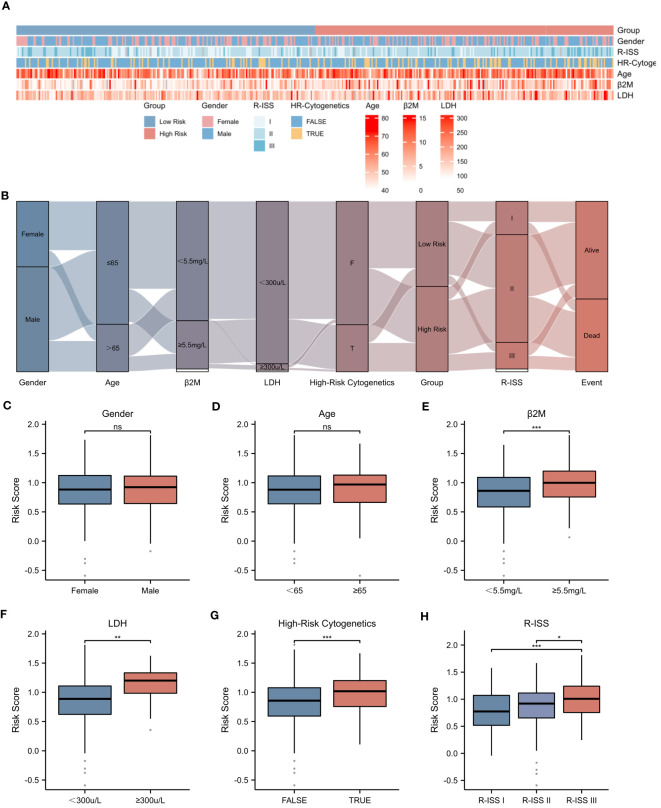
Correlation between Risk Score and Clinical Characteristics. **(A)** Heatmap showing the complexity of high-risk/low-risk groups, clinical characteristics, and expression levels of six genes. **(B)** Sankey diagram illustrating the relationship between clinical information and high-risk/low-risk groups. **(C-H)** Associations between risk score and different clinical features: **(C)** Gender. **(D)** Age. **(E)** β2-Microglobulin levels (β2M ≥ 5.5mg/L). **(F)** Lactate dehydrogenase levels (LDH ≥ 300μ/L). **(G)** High-Risk Cytogenetics. **(H)** R-ISS stage. Asterisks indicate statistical significance: *p < 0.05; **p < 0.01; ***p < 0.001; ns, no statistical signifcance.

The prognosis of MM patients is currently assessed using the International Staging System (ISS), the Revised International Staging System (R-ISS), and the mSMART risk stratification system. However, there remains heterogeneity in patient outcomes despite these assessments. In the dataset GSE136337, we found that it contains rich clinical data of MM patients. Utilizing the criteria established in mSMART 3.0 (2018), we defined high-risk cytogenetics in multiple myeloma as follows: deletion of the short arm of chromosome 17 [del(17p)], translocation between chromosomes 4 and 14 [t (4;14)], translocation between chromosomes 14 and 16 [t (14;16)], translocation between chromosomes 14 and 20 [t (14;20)], and gain of the long arm of chromosome 1 [1q+]. The heatmap presented in **Figure 5A** illustrates the correlation between the risk score and various patient-specific factors, including gender, age, β2M level, LDH level, R-ISS stage, and the presence of high-risk cytogenetics within the GSE136337 dataset. Notably, the heatmap reveals that patients in the high-risk group tended to have higher R-ISS stages, elevated β2M levels, and increased LDH levels. Furthermore, the Sankey diagram depicted in **Figure 5B** describes the relationships between patient gender, age, β2M level (≥5.5mg/L), LDH level (≥300μ/L), high-risk cytogenetics, R-ISS stage, high-risk/low-risk classification, and prognosis. Our analysis showed that most patients with high-risk cytogenetics were categorized in the high-risk group, while the majority of patients in the low-risk group were in the earlier ISS-I and ISS-II stages, often associated with a favorable prognosis. However, most patients in the high-risk group were found in the ISS-II and ISS-III stages, typically correlating with a poor prognosis. Additionally, we found that the NETs-related risk score was not associated with gender or age, as shown in **Figures 5C, D**. Importantly, the risk score was significantly correlated with tumor burden (elevated LDH and β2M levels), high-risk cytogenetic abnormalities, and advanced R-ISS stages (p < 0.01), as demonstrated in **Figures 5E–H**.These findings highlight the significant disparities presented by a NETs-based risk score system within the prognostic factors for patients with multiple myeloma, underlining the importance of risk score in stratifying the prognosis of multiple myeloma patients.

### 3.5 Construction of a nomogram based on risk score

**Figure 6:**
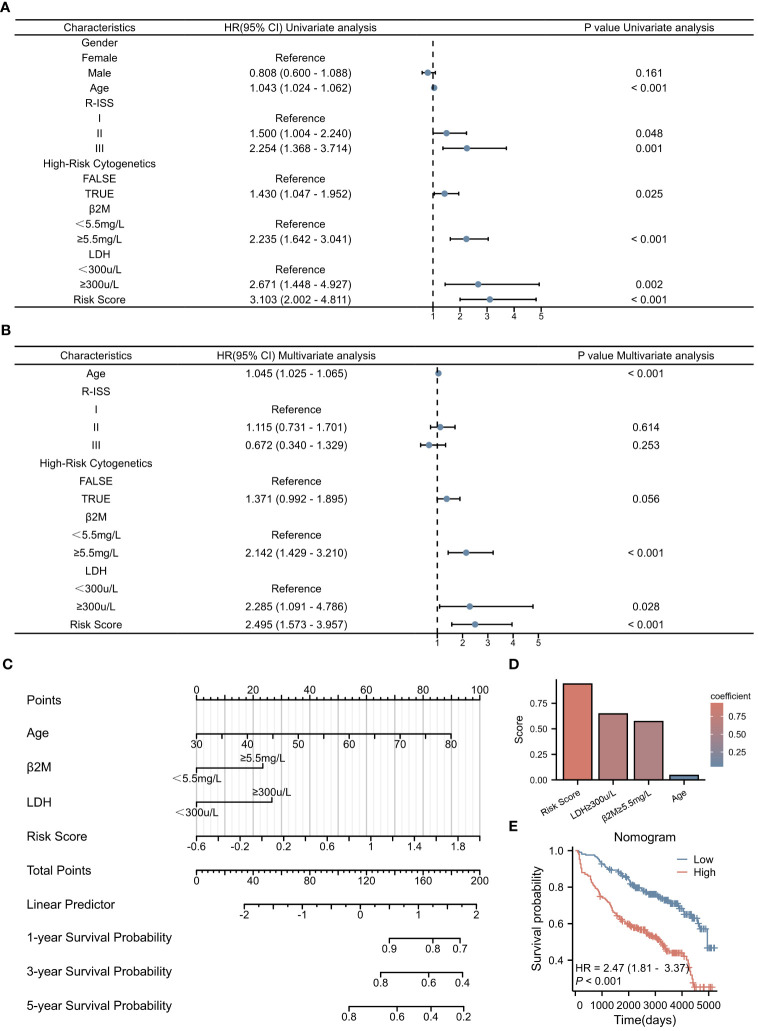
Construction of Prognostic Model Based on Risk Score. **(A)** Univariate Cox regression analysis of clinical parameters and the risk score. **(B)** Multivariate Cox regression analysis of clinical parameters and the risk score. **(C)** Nomogram displaying the scores for each factor, as well as the total score as a prognostic indicator. **(D)** Coefficients of each factor contributing to the total score. **(E)** Kaplan-Meier survival curves.

To rigorously assess the potential of the risk score as an independent prognostic factor, univariate and multivariate Cox regression analyses were conducted, encompassing a range of clinical parameters alongside the risk score (**Figures 6A, B**). Our findings unequivocally established that age, β2M level ≥ 5.5 mg/L, LDH level ≥ 300 μ/L, and the risk score itself emerged as independent prognostic indicators for MM patients. Utilizing a nomogram approach, we assigned specific scores to each of these factors and aggregated them to derive a comprehensive prognostic score (**Figure 6C**). The coefficients assigned to each factor, as showcased in **Figure 6D**, revealed that the risk score carried the highest weightage, thereby making a substantial contribution to the overall prognostic score. The Kaplan-Meier survival analysis further corroborated the nomogram’s efficacy in stratifying patients based on their OS, exhibiting statistical significance (p < 0.001). This underscores the nomogram’s potential as a valuable tool in predicting survival outcomes for MM patients.

### 3.6 Assessment of the nomogram

**Figure 7:**
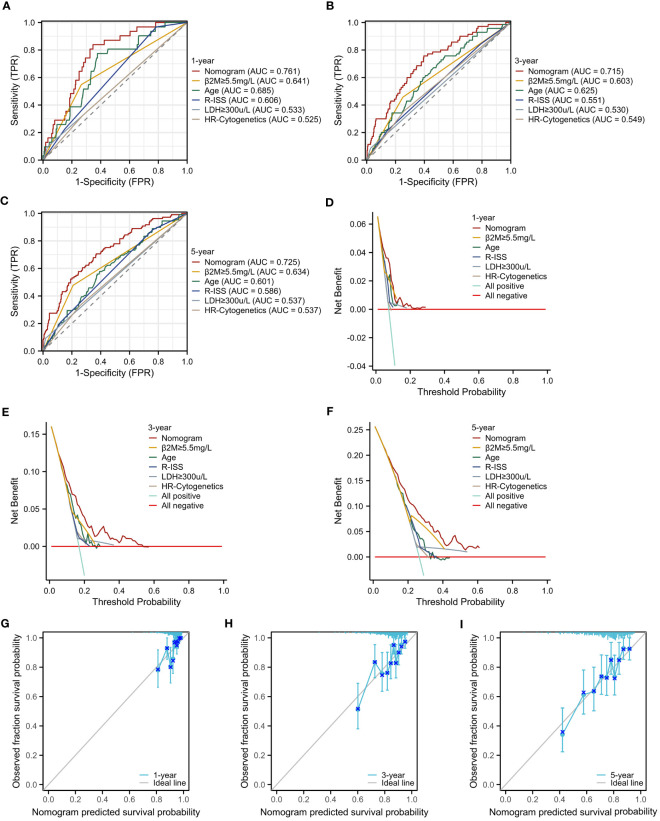
Assessment of the Prognostic Model. **(A–C)** ROC curves for overall survival over time for the prognostic model, ISS, β2M, and age: **(A)** 1 year, **(B)** 3 years. **(C)** 5 years. **(D–F)** Decision curve analysis (DCA) for the prognostic model, ISS, β2M, and age: **(D)** 1 year, **(E)** 3 years, **(F)** 5 years. **(G–I)** Calibration curves for the prognostic model: **(G)** 1 year, **(H)** 3 years, **(I)** 5 years.

To rigorously evaluate the prognostic model’s performance, we undertook a comprehensive and detailed assessment. Initially, we generated ROC curves for 1-, 3-, and 5-year survival predictions (**Figures 7A–C**). These results revealed that the nomogram surpassed the predictive abilities of R-ISS, β2M level ≥ 5.5 mg/L, LDH level ≥ 300 μ/L, high-risk cytogenetics, and age, achieving AUC values of 0.761, 0.715, and 0.725 for 1-, 3-, and 5-year OS, respectively. Subsequently, we employed decision curve analysis to assess the clinical utility of our predictive model across varying threshold probabilities. This analysis demonstrated that our prognostic model conferred the highest net benefit within specified threshold probability ranges for 1-, 3-, and 5-year predictions when compared to R-ISS, β2M, and age (**Figures 7D–F**). Furthermore, to validate the agreement between observed outcomes and the model’s predictions, we constructed calibration curves for 1-, 3-, and 5-year predictions (**Figures 7G–I**). These curves exhibited a close alignment of the predicted lines with the diagonal, indicating excellent calibration and reinforcing our model’s ability to make accurate predictions. The results validated the effectiveness of the nomogram in predicting survival may better than the R-ISS, β2M, LDH, high-risk genetic abnormalities and age, among other factors.

### 3.7 Evaluation of immune microenvironment in high- and low-risk groups

**Figure 8:**
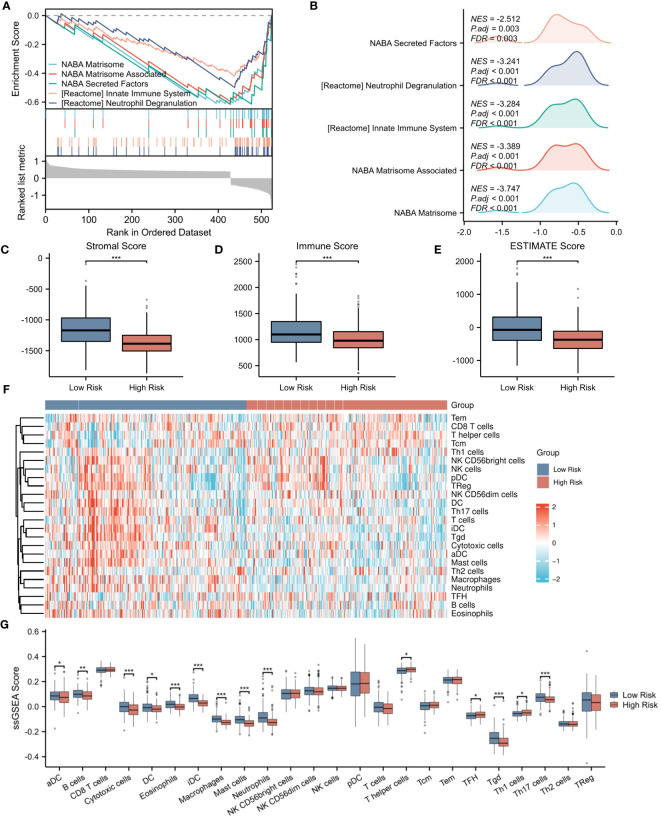
Evaluation of immune microenvironment in high- and low-risk groups. **(A)** GSEA enrichment analysis of differentially expressed genes in the high- and low-risk groups. **(B)** Landscape of GSEA enrichment for differentially expressed genes in the high- and low-risk groups. **(C–E)** Comparison of Stromal Score, Immune Score, and ESTIMATE Score between the high- and low-risk groups. **(F)** Expression heatmap for 23 types of immune cells in the high- and low-risk groups. **(G)** Differences in the expression levels of 23 immune cell types between the high- and low-risk groups. *p < 0.05; **p < 0.01; ***p < 0.001.

To further elucidate the underlying mechanisms associated with the NETs-related risk score, we conducted a GSEA focusing on DEGs in both high-risk and low-risk groups. The results, presented in **Figures 8A, B**, reveal a significant enrichment of these genes in pathways related to “secreted factors,” “neutrophil degranulation,” and the “innate immune system” (p < 0.01). These pathways are intimately associated with the formation of NETs and the modulation of immune function, suggesting that the disparity in gene expression between the high-risk and low-risk groups may reflect divergent immunological profiles. Given the observed enrichment of DEGs in immune-related pathways in both risk groups, we proceeded to explore the relationship between the risk score and immune status. Utilizing the ESTIMATE algorithm for a more in-depth analysis, we found that patients in the low-risk group exhibited significantly higher stromal scores (assessing the presence of non-malignant stromal cells in tumors), immune scores (quantifying the infiltration of immune cells in tumors), and ESTIMATE scores (comprehensively evaluating the tumor microenvironment, including the influence of stromal and immune cells) compared to those in the high-risk group (**Figures 8C–E**).To further validate this observation, we employed the ssGSEA algorithm to analyze the expression of tumor-infiltrating immune cells. The results, presented in **Figure 8F**, demonstrate a significant reduction in the presence of cytotoxic cells, eosinophils, immature dendritic cells (iDC), macrophages, mast cells, neutrophils, Tγδ (Tgd) cells, and Th17 cells in the high-risk group (p < 0.001) (**Figure 8G**). These immune cells play crucial roles in anti-tumor immune responses, and their reduced presence in the high-risk group may contribute to a weakened immune response to tumors, thereby affecting patient prognosis.

**Figure 9:**
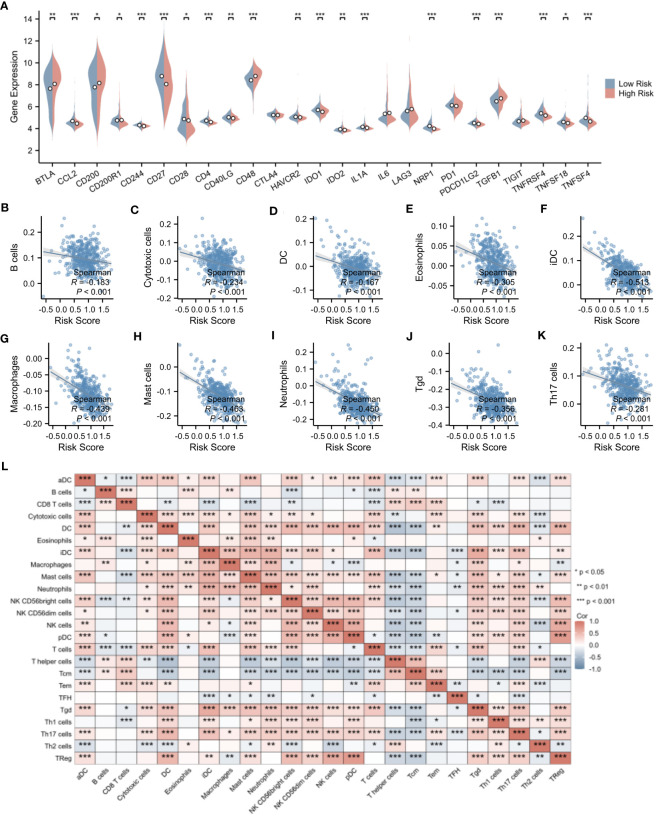
Evaluation of immune microenvironment in high- and low-risk groups. **(A)** Differences in the expression levels of immune checkpoints between the high- and low-risk groups. **(B–K)** Correlation analysis of risk scores with the expression levels of immune cells. **(L)** Correlation analysis among immune cell types. *p < 0.05; **p < 0.01; ***p < 0.001.

Delving deeper into the disparities in the expression of pivotal immune molecules between the high- and low-risk groups, we obtained notable findings as depicted in **Figure 9A**. Through meticulous analytical comparisons, a conspicuous downregulation trend emerged in the expression of conventional immune checkpoint molecules in the high-risk group relative to the low-risk counterparts. Specifically, a range of crucial molecules, namely CCL2, CD244, CD27, CD4, IDO1, IL1A, NRP1, PDCD1LG2, TNFRSF4, and TNFSF4, exhibited significantly reduced expression levels in the high-risk group. Conversely, a marked upregulation was observed in the expression of CD48 and TGFβ1 in the high-risk group (p < 0.001), presenting potential targets for future immunotherapeutic strategies. To shed further light on the relationship between risk scores and immune cell types, we conducted extensive correlation analyses. The results revealed a significant negative correlation between risk scores and the infiltration levels of the majority of immune cell types. Notably, a statistically significant negative correlation was observed between risk scores and the infiltration of B cells, cytotoxic cells, DC, eosinophils, immature dendritic cells (iDCs), macrophages, mast cells, neutrophils, helper T cells, Tγδ cells, and Th17 cells (p < 0.001). This finding is vividly illustrated in **Figures 9B–K**. Moreover, we explored the interrelationships among immune cells. Intriguingly, a significant positive correlation was uncovered between iDCs and mast cells (p < 0.001), whereas a marked negative correlation was observed between helper T cells and dendritic cells (p < 0.001). **Figure 9L** clearly presents these correlation analysis results among immune cells, unveiling the intricate network of interactions within the immune system.

### 3.8 Prediction of potential drugs for multiple myeloma based on nets-related risk score

**Figure 10:**
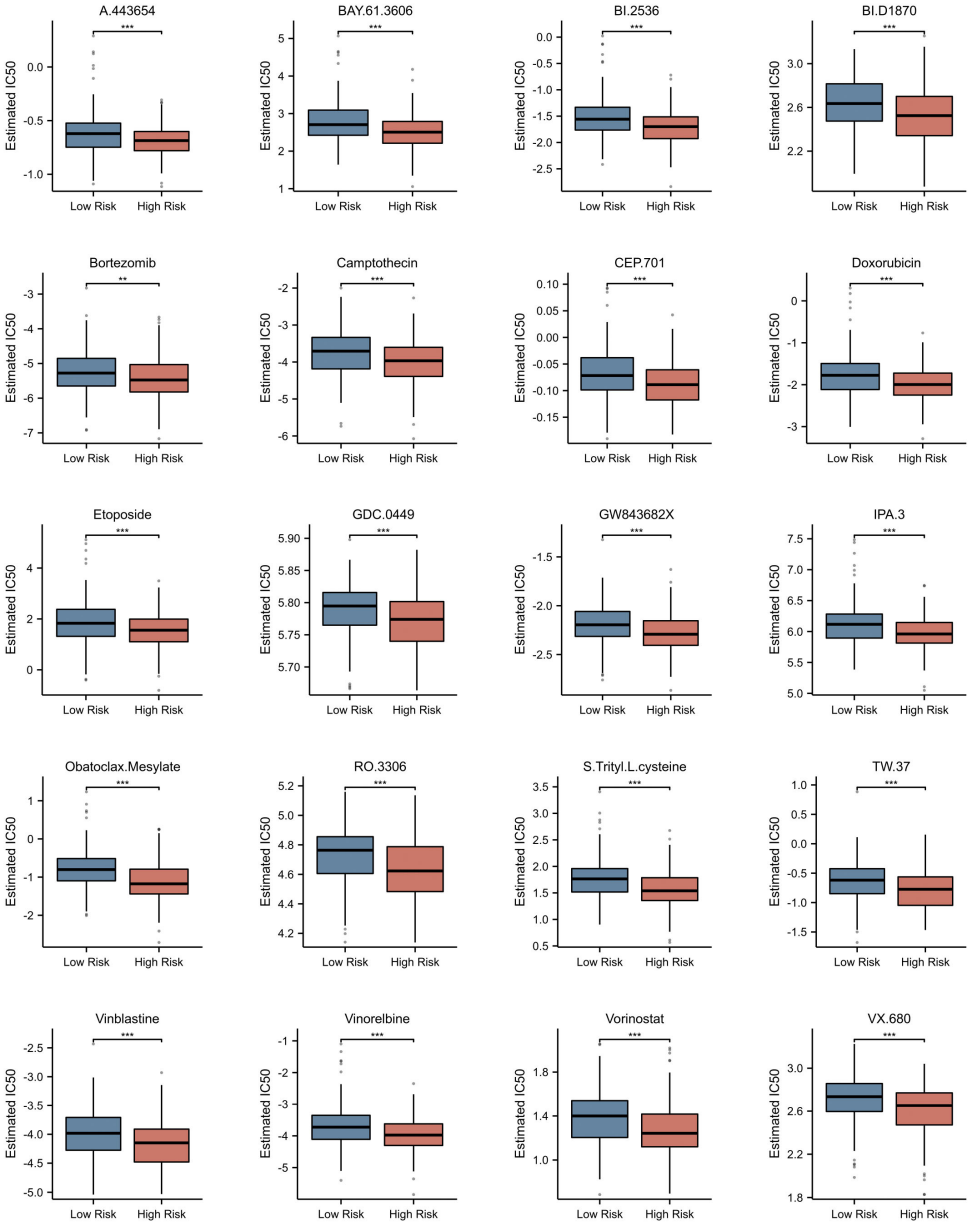
Prediction of Potential Drugs for Multiple Myeloma Based on NETs-related Risk Score. Estimated IC50 of Different Drugs in High and Low Risk Groups. **p<0.01; ***p<0.001.

Utilizing the pRRophetic package, we conducted a comprehensive investigation into the differences in estimated IC50 values of chemotherapeutic agents between high-risk and low-risk patient groups (detailed data presented in **Supplementary Table 4**). This algorithm, grounded in genomic data, provided us with valuable predictions of drug responses. Through statistical analysis, we uncovered a notable finding: the widely used drug for multiple myeloma treatment, bortezomib, exhibited a significant difference in estimated IC50 values between the high-risk and low-risk patient groups (p<0.001). Remarkably, this trend was not isolated to bortezomib alone. A range of other chemotherapeutic agents, including A.443654, BAY.61–3606, BI.2536, BI.D1870, camptothecin, CEP.701, doxorubicin, etoposide, GDC.0449, GW843682X, IPA.3, obatoclax mesylate, RO.3306, S-trityl-L-cysteine, TW.37, vinblastine, vinorelbine, vorinostat, and VX.680, also demonstrated a similar pattern (**Figure 10**). Specifically, for these drugs, the estimated IC50 values were significantly lower in the high-risk group compared to the low-risk group (p<0.0001). This discovery carries profound clinical implications. It suggests that patients with higher risk scores may derive greater therapeutic benefits from these chemotherapeutic agents.

### 3.9 qRT-PCR

**Figure 11:**
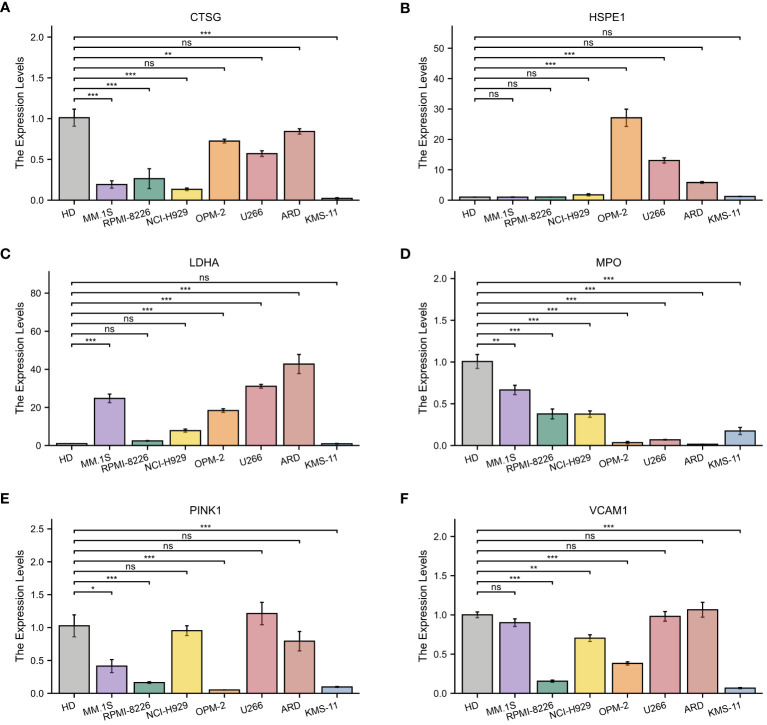
Quantitative Real-Time Polymerase Chain Reaction Analysis. **(A–F)** In MM cell lines and healthy donors: **(A)** CTSG; **(B)** HSPE1; **(C)** LDHA; **(D)** MPO; **(E)** VCAM1; **(F)** PINK1. *p<0.05; **p<0.01; ***p<0.001; ns, no statistical signifcance.

Subsequently, the expression of the identified genes was experimentally verified in MM cell lines MM.1S, RPMI-8266, NCI-H929, OPM-2, U266, ARD, KMS-11, and the healthy donors using qRT-PCR. As anticipated, the levels of CTSG, MPO, PINK1, and VCAM1 were lower in the MM cell lines compared to the control group, while HSPE1 and LDHA showed significantly higher expression in the MM cells (**Figure 11**).

## 4 Discussion

Multiple Myeloma is an incurable plasma cell neoplasm. Extensive literature has demonstrated that the resistance to treatment and disease progression in MM largely depends on the interactions between MM cells and the components of the bone marrow microenvironment (25). Neutrophils are generated in great number in the bone marrow, as a crucial part of the bone marrow microenvironment, release NETs which may influence the bone marrow tumor immune microenvironment and, in turn, affect the prognosis of patients with myeloma. NETs have been implicated in a variety of cancerous disease (26–31). However, the relationship between NETs and MM has not been reported.

In this study, we first combined NETs associated with MM survival to construct a risk score comprising six genes. Patients were divided into high-risk and low-risk groups based on the risk score, with the high-risk group displaying shorter OS. Subsequently, we developed a nomogram incorporating clinical indicators. The six genes we selected include CTSG, HSPE1, LDHA, MPO, PINK1, and VCAM1. Among these, CTSG is a gene of serine protease, which can control the effector function of adhesion-dependent neutrophils by modulating integrin clustering (32) and is involved in destroying intracellular and extracellular pathogens through nonoxidative pathways (33). Studies have confirmed that NETs promote endothelial cell activation and increase thrombosis through the synergistic action of IL-1α and CTSG (21). There have been reported that high expression of CTSG can inhibit the progression of colorectal cancer (34) and oral squamous cell carcinoma (35). Our results indicated that CTSG expression was lower in MM patients compared to normal individuals, and that high-risk MM patients expressed much lower CTSG than low-risk ones. HSPE1 is a heat shock protein. HSPE1 was associated with obesity, inflammation and NETs release (36). HSPE1 is a kind of heat shock protein. Studies have confirmed that HSPE1 is highly expressed in the leukemia stem cells of childhood AML (37), and other research indicated that upregulation of HSPE1 promoted prostate cancer progression (38). Our results showed that high expression of HSPE1 was detrimental to the prognosis of MM patients and was increased in MM patients compared to normal individuals. LDHA is an enzyme involved in glycolytic metabolism, primarily catalyzing the conversion of pyruvate to lactate in the cytoplasm. Neutrophil acidity through increasing LDHA activity enhances neutrophil migration *in vivo* and *in vitro* (19), and notably, in our risk scoring, LDHA was the molecule with the most unfavorable prognosis. Previous studies have found LDHA could promote the progression of lung cancer (39), lung adenocarcinoma (40), pancreatic cancer (41), and renal clear cell carcinoma (42). MPO is a myeloperoxidase, which plays an important role in the formation of NETs, promoting the autonomic formation of NETs and is considered a representative marker of NETosis (43). One of the main functions of MPO is to kill microorganisms in phagocytes, and MPO can also be released outside the cell to destroy various target substances (44). MPO plays a dual role in tumor progression (45). Our results suggested that the expression of MPO gradually was decreased with the different stages of plasma cell disease, and high-risk patients expressed much lower levels of MPO than low-risk ones. Infections are a common complication in patients with multiple myeloma, and high expression of MPO might prevent severe infections in individuals with multiple myeloma. MPO has been proven to have therapeutic potential for pathological bone loss mediated by osteoclasts (46), potentially improving the adverse prognosis of MM patients due to bone disease. In addition, MPO could induce the activation of caspase-3 and apoptosis in HL-60 human leukemia cells (47). PINK1 is a key protein involved in mitochondrial autophagy. NETs increase the expression of the mitochondrial autophagy-related protein PINK1 (48), and PINK1-dependent mitochondrial autophagy reduction was related to shortened OS and event-free survival in MM patients (49). Jia et al., through bioinformatics analysis, also confirmed PINK1 as a protective gene in MM (50). VCAM1 is a cell adhesion molecule. NETs could induce the expression of adhesion molecules in human endothelial cells and increase the adherence of white blood cells to the monolayer (21). Circulating levels of VCAM1 were also elevated in patients with venous thromboembolism, indicating not only exacerbated endothelial activation and dysfunction but also the favorable interaction of neutrophil adhesion molecules with their endothelial ligands for neutrophil migration. Previous studies have shown that increased myeloma cells can promote their extravasation and retention in the bone marrow through interactions with endothelial interaction molecules such as VCAM1 (51), suggesting that VCAM1 plays a role in promoting cancer survival and growth in MM, consistent with our results. Studies also indicated a reduction in VCAM1 expression in AML could reduce leukemic stem cell infiltration (52). In summary, the expression levels of these six NETs-related genes vary across different cancers, reflecting the extreme complexity of their potential regulatory mechanisms. Therefore, it becomes particularly important and urgent to further explore the biological characteristics of these genes in multiple myeloma.

Previous *in vitro* studies have identified the antitumor effects of NETs in melanoma (53), NETs can also reduce the proliferation of AML cells (54). However, some studies suggested that neutrophil extracellular traps enhanced the metastatic potential of hepatocellular carcinoma (55), non-small cell lung cancer (56), and others. Therefore, the overall activity of NETs, that either improves anti-tumor response or pro-tumor, probably depends on the complex interaction among the tumor cells, the NETs and the TME (57). GSEA based on the DEGs between high- and low-risk groups reveals significant enrichment in pathways immune system. Our study validated that anti-tumor immune activity is significantly reduced in high-risk MM patients with a high-risk score. The analysis of risk scores and immunity may partially explain the poor prognosis in the high-risk group: fewer immune cells are present in this group, resulting in worse outcomes compared to the low-risk group.

Routine immune checkpoint and drug sensitivity analysis provide potential therapeutic strategies for high-risk MM patients. Our analysis showed that CD48 and TGFβ1 were significantly upregulated in the high-risk group (p < 0.001), serving as potential therapeutic targets for this group. CD48 is expressed only on certain hematopoietic stem/progenitor cells and is not present on red blood cells or platelets, which has already been confirmed as a novel molecular target for antibody therapy in multiple myeloma (58, 59). TGFβ1 is a multifunctional cytokine that plays a pivotal role in hematopoiesis, tumor development, and immune regulation. Previous studies have indicated that targeting immunosuppression by TGFβ1 is a viable strategy for cancer immunotherapy (60). Interestingly, in MM, inhibition of TGFβ1 could counteract the growth advantages conferred by the adherence of MM cells to bone marrow stromal cells, as well as cytokine production in the bone marrow microenvironment, and enhance the host’s anti-MM immunity (61). Additionally, our analysis predicted that high-risk MM patients may be more sensitive to bortezomib, which is a frontline maintenance therapy drug for high-risk MM patients. Among the six genes we have screened, studies have indicated that knockdown of LDHA can restore sensitivity of bortezomib resistance cell lines while gain-of-function studies using LDHA induced resistance in bortezomib-sensitive cell lines (62). Our study results provided new insights into novel treatment methods for MM. Lastly, we discovered that the six genes we identified can not only predict the prognosis of MM patients but may also play a role in the different stages of plasma cell disorders.

Our study also has some limitations. Firstly, this research relied on RNA expression profiles and related clinical information downloaded from databases, the conclusions of which have not yet been validated in an actual clinical setting. Secondly, due to the lack of comprehensive clinical information from two independent datasets, our prognostic model has not been confirmed through a validation cohort. Lastly, the prognostic model was developed based on retrospectively collected data from public databases; its accuracy and reliability require further confirmation through real-world data and prospective studies. We intend to collect more clinical datasets to reaffirm the significance of these NETs-related genes.

## 5 Conclusion

In conclusion, our study demonstrated the correlation between NETs and MM, suggesting a potential role of NETs in the pathogenesis of MM. Furthermore, using expression levels of six genes from newly diagnosed patients’ bone marrow, we calculated risk scores predictive of prognosis. High-risk patients had shorter OS, fewer immune cells, and worse clinical markers. We also predicted drug responses in this group to aid clinical decision-making. While further research is needed to validate and expand upon our observations, our report on the correlation between NETs and MM may provide new insights for future research and the development of improved clinical management of MM.

## Data availability statement

The original contributions presented in the study are included in the article/**Supplementary Material**, further inquiries can be directed to the corresponding author/s.

## Ethics statement

In compliance with the 1964 Helsinki Declaration and its subsequent amendments, or equivalent ethical guidelines, all participants willingly gave their written consent to be involved in this research study.

## Author contributions

GG: Conceptualization, Data curation, Formal analysis, Investigation, Methodology, Project administration, Software, Supervision, Writing – original draft. RL: Conceptualization, Data curation, Formal analysis, Investigation, Methodology, Software, Validation, Writing – original draft. DW: Data curation, Formal analysis, Investigation, Methodology, Validation, Writing – original draft. GD: Data curation, Formal analysis, Investigation, Methodology, Validation, Writing – original draft. YL: Formal analysis, Writing – original draft. XX: Formal analysis, Writing – original draft. BF: Formal analysis, Writing – original draft. ZL: Formal analysis, Writing – original draft. TW: Formal analysis, Writing – original draft. AH: Conceptualization, Supervision, Validation, Writing – review & editing. JB: Conceptualization, Supervision, Validation, Writing – review & editing.
