# Supplementary figures and images for: Risk score constructed with neutrophil extracellular traps-related genes predicts prognosis and immune microenvironment in multiple myeloma
Source: Front Oncol. 2024 Jun 11;14:1365460. doi: 10.3389/fonc.2024.1365460 (PMC11196624; doi:10.3389/fonc.2024.1365460)

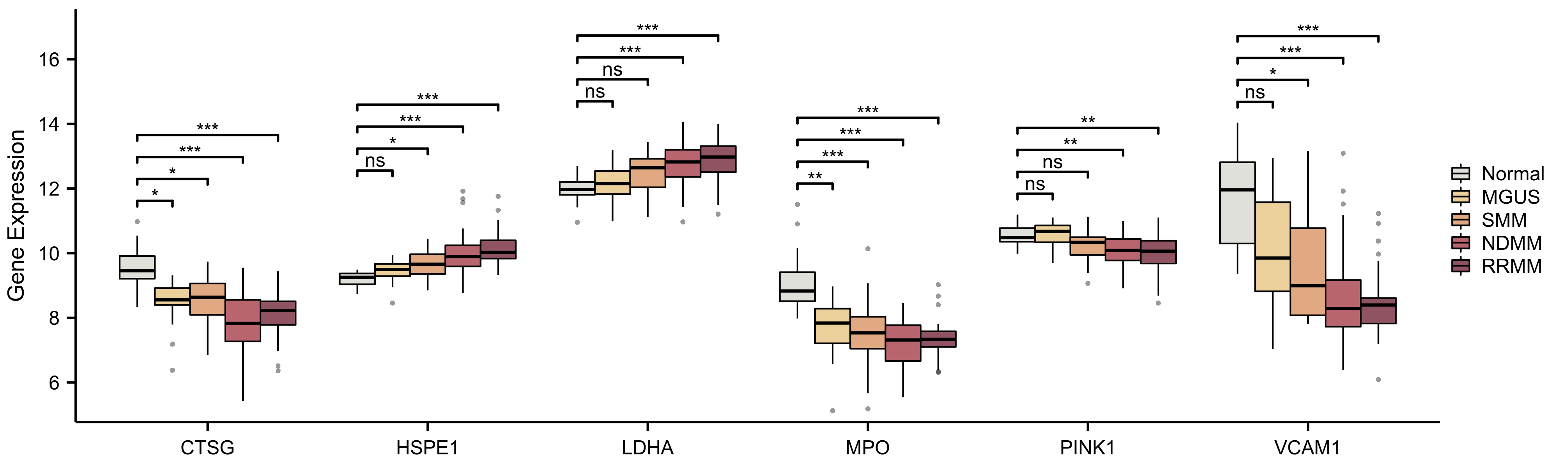

Supplement: Supplementary file 1 [file Image_1.tiff]

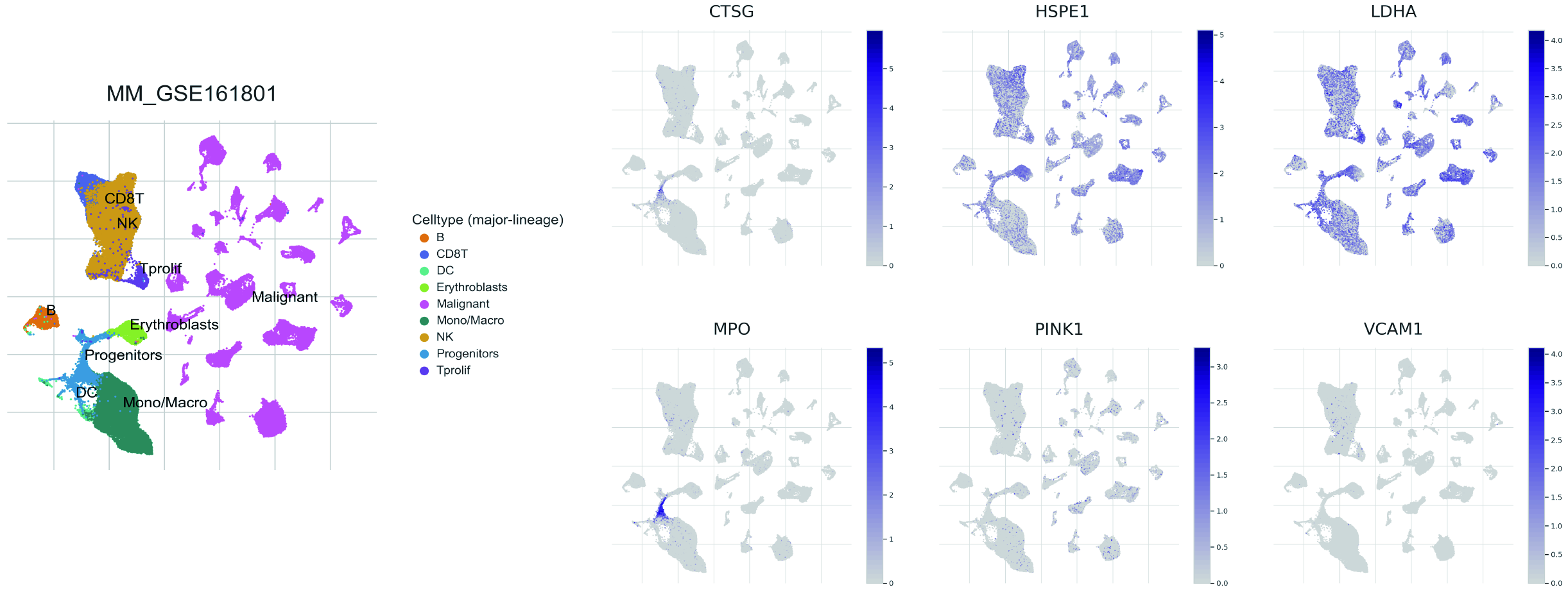

Supplement: Supplementary file 2 [file Image_2.tif]
